# Masked Hypotension due to Elevated Venous Pressure in a Patient with Complex Adult Congenital Heart Disease

**DOI:** 10.1155/2020/7148708

**Published:** 2020-03-31

**Authors:** Wytch Rigger, Sean P. Javaheri, Gyanendra K. Sharma, Norris Stanley Nahman, Mark Sheynshteyn, Neal L. Weintraub

**Affiliations:** ^1^Department of Medicine, Medical College of Georgia at Augusta University, Augusta, GA, USA; ^2^Division of Cardiology, Medical College of Georgia at Augusta University, Augusta, GA, USA; ^3^Division of Nephrology, Medical College of Georgia at Augusta University, Augusta, GA, USA; ^4^Division of Endocrinology, Diabetes and Metabolism, Medical College of Georgia at Augusta University, Augusta, GA, USA

## Abstract

An adult with surgically corrected Tetralogy of Fallot presented with profoundly elevated central venous pressure (CVP) and acute renal dysfunction thought secondary to acute on chronic right heart failure. Treatment with dopamine promoted diuresis and a stabilization of renal function. Repeated attempts to wean the patient from dopamine were associated with hypotension and worsening renal failure. Invasive hemodynamic assessment unexpectedly demonstrated high cardiac output with low systemic vascular resistance (SVR). In retrospect, the markedly elevated CVP had concealed the impact of reduced SVR on blood pressure. After reversible causes of low SVR state were excluded, the patient was successfully managed with oral alpha-adrenergic agents. While typically negligible under physiologic conditions, elevated CVP can artificially increase mean arterial pressure. We have coined the term “masked hypotension” to describe this unique pathophysiological phenomenon.

## 1. Introduction

Due to advances in surgical techniques, the population of adults with congenital heart disease is growing [1]. As these patients age, understanding the interplay between their congenital heart disease and acquired diseases is imperative to providing comprehensive care. We report the case of an adult with Tetralogy of Fallot who presented with what appeared to be right-sided heart failure, only to discover that the patient was actually in a high cardiac output state. Management required an acute understanding of his unique hemodynamic physiology and the different determinants of tissue perfusion pressure.

## 2. Case

**Figure 1:**
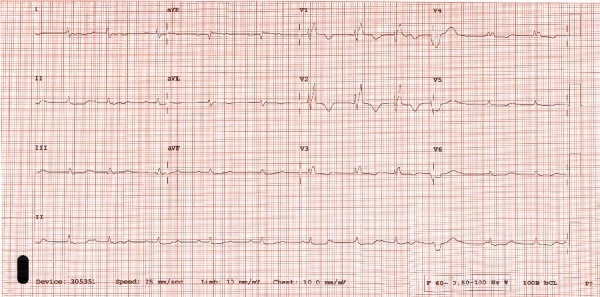
Electrocardiogram on presentation. The patient was noted to be in sinus rhythm with a first-degree AV block with a prolonged PR interval of 223 msec and a stable right bundle branch block that had been present for many years.

A 61-year-old male with a history of surgically corrected Tetralogy of Fallot presented with fatigue, edema, weight gain, and worsening renal insufficiency. He exhibited marked jugular venous distension, ascites, and edema on examination. Historically, he had undergone complete surgical repair at age 11, a right ventricular outflow tract reconstruction and pulmonary valve replacement at age 47, and a percutaneous pulmonary valve replacement at age 59. In the months prior to admission, the patient exhibited worsening signs and symptoms of volume overload requiring escalating diuretic doses. Prior to admission, a cardiac MRI demonstrated marked biatrial enlargement, right ventricular dilation and dysfunction, and moderate pulmonary and tricuspid regurgitation (Supplemental [Supplementary-material supplementary-material-1]). An echocardiogram on admission demonstrated right ventricular dilation with an estimated right ventricular systolic pressure of 54 mmHg, moderate tricuspid regurgitation, and mild pulmonary conduit stenosis. Electrocardiogram at the time of admission demonstrated sinus rhythm with a stable right bundle branch block (Figure 1).

**Figure 2:**
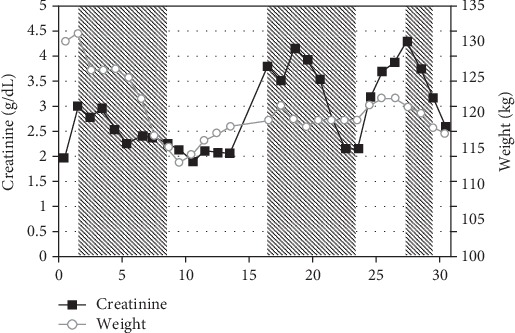
Plot of serum creatinine and weight over the 30 days after initial presentation. Shaded areas represent time periods where the patient received treatment with intravenous vasopressor agents. Dopamine was used as the vasopressor on days 1-8 and 16-23 while intravenous phenylephrine was used on days 27-29 with the patient being transitioned to oral phenylephrine thereafter. Note that despite correction of volume status by day 10, and the relative stability of weight thereafter, the patient still developed renal failure (second shaded area) supporting a pathophysiologic role for distributive shock in the face of high-output cardiac failure.

The patient was admitted for treatment of presumed decompensated right heart failure. MAP was 64 mmHg, and CVP was markedly elevated at 27 mmHg. He was started on intravenous diuretics and dopamine and successfully diuresed over 12 days to a negative fluid balance of 16.5 L with an improvement in renal function (Figure 2).

**Figure 3:**
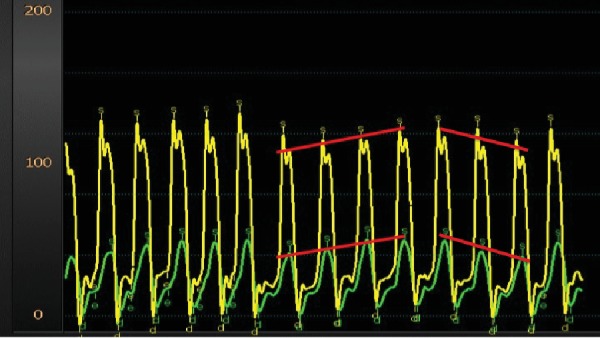
Simultaneous left and right ventricle hemodynamic monitoring with left ventricular pressure shown in yellow and right ventricular pressure shown in green. Red lines added to highlight the concordance between right ventricular and left ventricular pressure with respiration. Concordance between right ventricular and left ventricular pressures with respiration is suggestive of restrictive physiology.

Three days after discharge, the patient was readmitted with frank hypotension, hyperkalemia, and oliguric renal failure. Dopamine infusion was resumed with improvement in renal function and urine output. Repeated attempts to wean the dopamine were associated with dramatic drops in blood pressure and oliguric renal failure (Figure 2). Simultaneous right and left heart catheterization performed off dopamine surprisingly demonstrated elevated CO and low SVR consistent with distributive shock (Table 1). Additionally, the patient was noted to have concordance of ventricular pressures that suggested of restrictive physiology (Figure 3). The pulmonary artery catheter was left in place, and subsequent measurements were made once the patient was placed back on dopamine (Table 1).

Potential reversible causes, including adrenal insufficiency and sepsis, were excluded. After a failed trial of dopamine weaning, a phenylephrine infusion was started. He was subsequently converted to oral phenylephrine and midodrine with stable hemodynamics, urine output, and renal function. Three weeks after discharge, he reported good exercise tolerance and urine output, with a stable creatinine.

On outpatient follow-up assessments, the patient remained stable on this medication regimen for several months until he suffered a mechanical fall at home and eventually died of complications related to this event.

## 3. Discussion

Initially, this patient's history and clinical features suggested a diagnosis of decompensated right heart failure, a common problem in adults with complex congenital heart disease. Therapy was initiated with diuretics and dopamine leading to improved symptoms and renal function, lending further credence to this diagnosis. After dopamine was withdrawn and he abruptly decompensated with hypotension and renal failure, a pulmonary artery catheterization led to the unexpected finding of high-output cardiac failure, low SVR, and a diagnosis of distributive shock. In retrospect, the markedly elevated CVP on presentation not only contributed to poor renal perfusion but also impeded the diagnosis of distributive shock in this patient. We have thus coined the term “masked hypotension” to describe this phenomenon.

Chronic high-output cardiac failure, characterized by an elevated CO and central venous congestion, is most often caused by anemia, thyrotoxicosis, obesity, hepatic disease, shunts, and COPD [2]. The cause of distributive shock was never fully explained in this case and was likely multifactorial. The patient was obese and had evidence of cirrhosis (not shown), although he did not exhibit signs or symptoms of advanced hepatic disease. Interestingly, his bilirubin level fell with dopamine infusion and diuresis (not shown), suggesting that liver dysfunction may have been a consequence, rather than a cause, of the high-output failure. Shunts are common in patients with adult congenital heart disease, but none was detected on catheterization (not shown). Other causes of distributive shock, including adrenal insufficiency, thyrotoxicosis, and sepsis, were excluded in this patient. Notably, patients with adult congenital heart disease were reported to exhibit elevated levels of endotoxin, which potentially could have contributed to low SVR in this patient [3].

Increased venous return from an elevated cardiac output alongside volume retention due to decreased renal perfusion pressure led to a dramatically elevated CVP. Under normal physiologic conditions, the contribution of CVP to MAP is negligible. However, markedly elevated CVP can significantly contribute to MAP [MAP = (SVR × CO) + CVP] and potentially “mask” arterial hypotension. Only after diuresis was it apparent that this patient was profoundly hypotensive and dependent on vasopressor agents to maintain MAP. It is not known if this phenomenon is unique to high-output failure. However, given that SVR is a fixed value and CO is maximally elevated at baseline, it would appear that CVP would be a greater determinant of MAP in high-output failure compared to other physiologic states.

In this patient, the profoundly elevated CVP hampered recognition of the low SVR state while contributing to impaired renal perfusion. While volume retention is an appropriate response to inadequate arterial filling pressures, the maladaptive increase in CVP contributed to the patient's development of cardiorenal syndrome. Elevated CVP has been shown to decrease GFR via multiple mechanisms, including a decrease in the transrenal arterial-venous pressure gradient as well as an increase in hydrostatic pressure within the renal capsule [4].

Dopamine was chosen initially in this case for several reasons. First, dopamine was reported to preserve renal function in some patients with acute heart failure exacerbation, although results have been mixed [5]. Second, the patient was presumed to have right heart failure, and dopamine has been suggested to improve right ventricular contractility without increasing pulmonary vascular resistance [6]. Third, the patient had marginally low MAP, so inotropes associated with vasodilatory properties (i.e., dobutamine and milrinone) were less desirable. In retrospect, it appears that the benefit derived from dopamine therapy was related solely to peripheral vasoconstriction mediated through alpha-adrenergic stimulation (Table 1), as validated by the patient's clinical response to oral phenylephrine.

The unique physiology of our patient highlights the challenge in managing adults with congenital heart disease; the prevalence of which is expected to grow due to increased survival from surgical advances [1]. In addition to cardiac disease, these patients have significantly elevated rates of extracardiac organ dysfunction compared to the general population [7]. Thus, understanding the altered physiology in adult congenital heart patients is paramount to proper diagnosis and management.

## 4. Conclusion

In summary, we present a case of distributive shock that was concealed by markedly elevated CVP in a patient with complex adult congenital heart disease. We have coined the term “masked hypotension” to describe this phenomenon. Proper diagnosis led to treatment with alpha-adrenergic agents, which stabilized his hemodynamic state and renal function.

## Figures and Tables

**Table 1:** Hemodynamic data obtained during right heart catheterization and bedside pulmonary artery catheter measurements.

	Without dopamine	With dopamine at 9 mcg/kg/min
MAP (mmHg)	45	94
CVP (mmHg)	14	20
Cardiac output (L/min)^∗^	9.05	7.46
CI (L/min/m^2^)	3.85	3.12
SVR (DS/cm^5^)	256	793
PAP (mmHg)	33/11 (21)	40/19 (27)
PCWP (mmHg)	16	20
PVR (DS/cm^5^)	35	75
RAP (mmHg)	12	n/a^#^
RVP (mmHg)	42/4 (12)	n/a^#^
LVP (mmHg)	113/-3 (16)	n/a^#^

^∗^Cardiac output calculated using Fick's formula. ^#^Direct measurements of right atrial pressure, right ventricular pressure, and left ventricular pressure were unable to be obtained using the bedside pulmonary artery catheter. CI = cardiac index; CVP = central venous pressure; MAP = mean arterial pressure; PAP = pulmonary artery pressure; PCWP = pulmonary capillary wedge pressure; SVR = systemic vascular resistance; RAP = right atrial pressure; RVP = right ventricular pressure; LVP = left ventricular pressure.

## Abbreviations

MAP: Mean arterial pressure
CVP: Central venous pressure
CO: Cardiac output
SVR: Systemic vascular resistance
GFR: Glomerular filtration rate.

## Supplementary Materials

Supplementary Materials Supplemental Figure 1: four chamber cardiac MRI cine images. Imaging notable for impaired right ventricular function with an ejection fraction of 35% (normal range 47-74%), diastolic septal wall flattening consistent with right ventricular volume overload, and severe right atrial dilation. These findings were suggestive of acute on chronic right heart failure as a cause of the patient's symptoms prior to invasive hemodynamic assessment.
